# Body Mass Index in Patients Treated with Cabozantinib for Advanced Renal Cell Carcinoma: A New Prognostic Factor?

**DOI:** 10.3390/diagnostics11010138

**Published:** 2021-01-18

**Authors:** Matteo Santoni, Francesco Massari, Sergio Bracarda, Giuseppe Procopio, Michele Milella, Ugo De Giorgi, Umberto Basso, Gaetano Aurilio, Lorena Incorvaia, Angelo Martignetti, Mimma Rizzo, Giacomo Cartenì, Enrique Grande, Marc R. Matrana, Simon J. Crabb, Nuno Vau, Giulia Sorgentoni, Alessia Cimadamore, Rodolfo Montironi, Nicola Battelli

**Affiliations:** 1 Oncology Unit, Macerata Hospital, Via Santa Lucia 2, 62100 Macerata, Italy; giulia.sorgentoni@libero.it (G.S.); nicola.battelli@sanita.marche.it (N.B.); 2 Division of Oncology, IRCCS Azienda Ospedaliero-Universitaria di Bologna, Via Albertoni 15, 40138 Bologna, Italy; fmassari79@gmail.com; 3 Medical and Translational Oncology Unit, Department of Oncology, AziendaOspedaliera Santa Maria, 05100 Terni, Italy; sergio.bracarda@gmail.com; 4 Department of Medical Oncology, Istituto Nazionale dei Tumori IRCCS, 20133 Milan, Italy; giuseppe.procopio@istitutotumori.mi.it; 5 Section of Oncology, Department of Medicine, University of Verona School of Medicine and Verona University Hospital Trust (AOUI Verona), P.le L.A. Scuro 10, 37134 Verona, Italy; michele.milella@univr.it; 6 Department of Medical Oncology, Istituto Scientifico Romagnolo per lo Studio e la Cura dei Tumori (IRST) IRCCS, 47014 Meldola, Italy; ugo_degiorgi@yahoo.com; 7 Department of Medical Oncology, Istituto Oncologico Veneto IOV IRCCS, 35128 Padova, Italy; umberto.basso@iov.veneto.it; 8 Medical Oncology Division of Urogenital and Head and Neck Tumours, IEO, European Institute of Oncology IRCCS, 20141 Milan, Italy; gaetano.aurilio@ieo.it; 9 Department of Biomedicine, Neuroscience and Advanced Diagnostics (Bi.N.D.), Section of Medical Oncology, University of Palermo, 90127 Palermo, Italy; lorena.incorvaia@unipa.it; 10 Dipartimento Oncologico USL Sud-Est Toscana-Area Senese, Località Campostaggia s.n.c., 53036 Poggibonsi, Italy; angelo.martignetti@uslsudest.toscana.it; 11 Division of Translational Oncology, IRCCS Istituti Clinici Scientifici Maugeri, 27100 Pavia, Italy; rizzo.mimma@gmail.com; 12 Department of Medical Oncology, AO “A. Cardarelli”, 80131 Naples, Italy; cartenigiacomo@gmail.com; 13 Department of Medical Oncology, MD Anderson Cancer Center, 28033 Madrid, Spain; egrande@mdanderson.es; 14 Department of Internal Medicine, Hematology/Oncology, Ochsner Medical Center, New Orleans, LA 70121, USA; mamatrana@ochsner.org; 15 Southampton Clinical Trials Unit, University of Southampton, Southampton SO16 5AF, UK; S.J.Crabb@southampton.ac.uk; 16 Urologic Oncology, Champalimaud Clinical Center, 1400-038 Lisbon, Portugal; nuno.vau@fundacaochampalimaud.pt; 17 Section of Pathological Anatomy, Polytechnic University of the Marche Region, School of Medicine, United Hospitals, 60126 Ancona, Italy; a.cimadamore@staff.univpm.it (A.C.); r.montironi@staff.univpm.it (R.M.)

**Keywords:** body mass index, cabozantinib, obesity, prognosis, real-world data, renal cell carcinoma, targeted therapy

## Abstract

We analyzed the clinical and pathological features of renal cell carcinoma (RCC) patients treated with cabozantinib stratified by body mass index (BMI). We retrospectively collected data from 16 worldwide centers involved in the treatment of RCC. Overall survival (OS) and progression-free survival (PFS) were analyzed using Kaplan–Meier curves. Cox proportional models were used at univariate and multivariate analyses. We collected data from 224 patients with advanced RCC receiving cabozantinib as second- (113, 5%) or third-line (111, 5%) therapy. The median PFS was significantly higher in patients with BMI ≥ 25 (9.9 vs. 7.6 months, *p* < 0.001). The median OS was higher in the BMI ≥ 25 subgroup (30.7 vs. 11.0 months, *p* = 0.003). As third-line therapy, both median PFS (9.2 months vs. 3.9 months, *p* = 0.029) and OS (39.4 months vs. 11.5 months, *p* = 0.039) were longer in patients with BMI ≥ 25. BMI was a significant predictor for both PFS and OS at multivariate analysis. We showed that a BMI ≥ 25 correlates with longer survival in patients receiving cabozantinib. BMI can be easily assessed and should be included in current prognostic criteria for advanced RCC.

## 1. Introduction

The American Cancer Society has estimated that there were a total of 73,750 new cases of kidney tumors (45,520 men and 28,230 women) in 2020 in the United States, with more than 14,000 cancer-related deaths [1]. Recently, we reported the results of an artificial neural networks (ANN) model to predict the incidence of renal cell carcinoma (RCC) in the United States for the future decades [2]. We showed that RCC incidence is expected to increase in the next years, thus supporting the necessity of more accurate studies on the prevention of RCC-related risk factors in order to reduce future tumor burden [2].

Beyond its well-known role as a risk factor for the development of RCC, obesity is also emerging as a potential key factor for response to therapy [3]. A growing body of evidence suggests that being overweight and obese are associated with better outcome in cancer patients treated with immunotherapy [4,5]. In this regard, Sanchez et al. investigated the angiogenic and immunologic transcriptomic profiles of the primary tumor and perinephric adipose tissue in normal weight and obese RCC patients. They reported that tumors from obese patients were enriched in the expression of vascular endothelial growth factor (VEGF) and related proteins. Moreover, a higher proportion of plasmacytoid dendritic cells (pDCs) and mast cells and a lower proportion of innate lymphoid cells (NK_CD56bright_cells) were observed in obese RCC patients [6]. In this context, leptin levels in obese subjects have been correlated to higher T cell programmed death (PD)-1 expression and improved response to anti-PD-1 therapy [7,8].

Cabozantinib is an orally administered tyrosine kinase inhibitor (TKI) acting mainly on VEGFR2 (VEGF receptor 2), MET (mesenchymal epithelial transition receptor), and AXL (anexelekto pathway) [9]. Currently, cabozantinib is approved for the treatment of patients with metastatic RCC in treatment-naïve adults with intermediate or poor-risk features and for adults that have progressed to prior vascular endothelial growth factor/receptor inhibitors. In 2019, we reported the results of an international retrospective real-world analysis on cabozantinib in previously treated patients with metastatic RCC, which was aimed at investigating the presence of prognostic factors in this context [10]. We observed that both hemoglobin (Hb) levels and International Metastatic Renal Cell Carcinoma Database Consortium (IMDC) prognostic models were associated with the outcome of patients receiving cabozantinib [10]. Furthermore, the median time to strategy failure (TTSF) was 11.57 months with the sequence cabozantinib–nivolumab and 25.64 months with nivolumab–cabozantinib [10].

Regarding the latter scenario, may we classify being overweight and obesity as eventual predictive factors of tumor response to immunotherapy in metastatic RCC? Hence, as a consequence, can we base our choice between cabozantinib and nivolumab as second- or third-line therapy on patient weight or body mass index (BMI)? To answer this question, we performed an international multicenter retrospective study to investigate BMI as a potential predictive factor of response in patients with advanced RCC receiving cabozantinib as second- or third-line therapy in respect mainly to nivolumab.

## 2. Materials and Methods

### 2.1. Study Population

We analyzed data from adult patients (aged 18 years and above) with a histologically confirmed diagnosis of RCC and histologically or radiologically confirmed metastatic disease treated with cabozantinib as second- or third-line therapy. Stage and grade were assigned by the local pathologist according to the American Joint Committee on Cancer (AJCC) TNM system and to the International Society of Urological Pathology (ISUP)/World Health Organization (WHO) grading system, respectively [11,12].

Standard biopsy procedures were followed according to the international European Association of Urology (EAU) guidelines [13]. This international multicenter retrospective study included data from sixteen institutions between 1 January 2008 and 1 October 2019. Data were retrospectively collected from paper and electronic charts. Patients were excluded from this study if they had missing data regarding the site of metastasis and response to therapy.

### 2.2. Treatment Regimens and Statistical Analysis

Cabozantinib was administered orally, mainly with a starting dose of 60 mg once daily. In patients with comorbidities, cabozantinib was initiated at a reduced dose of 40 mg once daily. Dose reductions and treatment interruptions were performed depending on the type and severity of adverse events according to standard guidelines. Treatment with cabozantinib was continued until the evidence of disease progression on computed tomography (CT) or magnetic resonance imaging (MRI) scans, unacceptable toxicity, or death. Follow-up commonly consisted of regular physical and laboratory assessment every 4–6 weeks. Imaging was carried out according to local procedures every 8–12 weeks.

Body mass index (BMI) was defined as weight expressed in kilograms divided by the square of the height in meters. Progressive disease was defined as a ≥20% increase in the sum of diameters of target lesions or by the appearance of one or more new lesions according to the Response Evaluation Criteria in Solid Tumors (RECIST) 1.1 criteria [14]. Progression-free survival (PFS) was defined as the time from the start of cabozantinib therapy to progression or to death from any cause. Patients without tumor progression or death at the time of the data cutoff were censored at their last follow-up date. Overall survival (OS) was defined as the time from the start of treatment to death from any cause. Patients alive or lost to follow-up were censored.

PFS and OS were estimated using the Kaplan–Meier method with Rothman’s 95% confidence intervals (CIs) and compared across the groups using the log-rank test.

Cox proportional hazards models were used to investigate patients’ characteristics and predictors of survival univariate and multivariate analyses. Chi-square test was used to compare categorical endpoints. All the significance levels were set at a 0.05 value and all *p* values were two-sided. The statistical analysis was performed by MedCalc version 11.4.4.0 (MedCalc Software, Broekstraat 52, 9030Mariakerke, Belgium). This project was performed in accordance with the approval by the ethical committees of our institutions.

## 3. Results

### 3.1. Study Population

We retrospectively collected data from 224 patients with advanced RCC receiving cabozantinib as second- (113, 5%) or third-line (111, 5%) therapy. The median age was 63 years (range 25–86). One hundred and sixty of the patients were males (71%). Tumor histology was predominantly clear cell (193, 9%); 51% of patients were metastatic at RCC diagnosis. Number of metastatic sites was ≥2 in 135 patients (60%). Lung (65%), lymph nodes (51%), and bone (28%) were the most common metastatic sites. According to IMDC criteria, 50 patients (22%) presented favorable-risk features, 134 (60%) presented intermediate-risk features, and 40 (18%) presented poor-risk features. The complete list of patients’ characteristics is reported in Table 1.

The median BMI was 26 (range 18–36). BMI was ≥25 in 119 patients (53%), where 15 patients (13%) had a BMI ≥ 30. Among the 105 patients with BMI < 25, 17 (16%) had a BMI ≤ 20. As reported in Table 1, no statistically significant differences in terms of demographic and disease characteristics were found between patients with BMI ≥ 25 and <25.

### 3.2. Response to Therapy and Survival Analysis

The median follow-up time from diagnosis was 182.79 months (95% CI 131.00 to not reached; NR). During the follow-up, 78 patients (35%) died. First-line therapy was sunitinib in 121 patients (54%), pazopanib in 73 (33%), and immunocombinations in 9 (4%). A total of 113 patients (50%) were treated with cabozantinib as second-line therapy, while 111 (50%) received cabozantinib as third-line therapy. In 21 patients (19%), second-line cabozantinib was ongoing at the time of data analysis. Among the 92 patients who progressed on second-line cabozantinib, 51 (55%) received a third-line therapy, which was nivolumab in 36 patients. Drug distribution is reported in Table 2.

**Figure 1:**
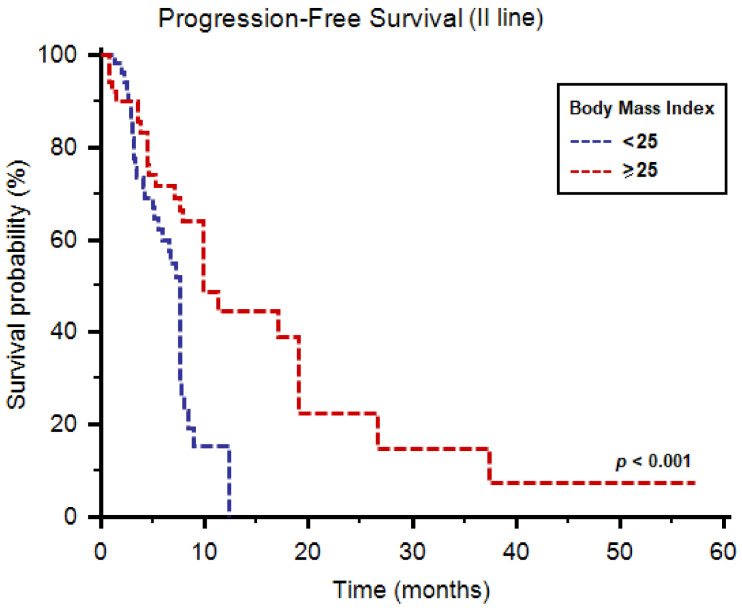
Progression-free survival of patients receiving cabozantinib as second-line therapy stratified by body mass index.

The median PFS of cabozantinib as second-line therapy was 7.8 months (95%CI: 7.6–9.9) in the overall study population. The median PFS was significantly higher in patients with BMI ≥ 25 (9.9 months, 95%CI: 7.9–19.1, vs. 7.6 months, 95%CI: 5.9–12.3, *p* < 0.001, Figure 1).

**Figure 2:**
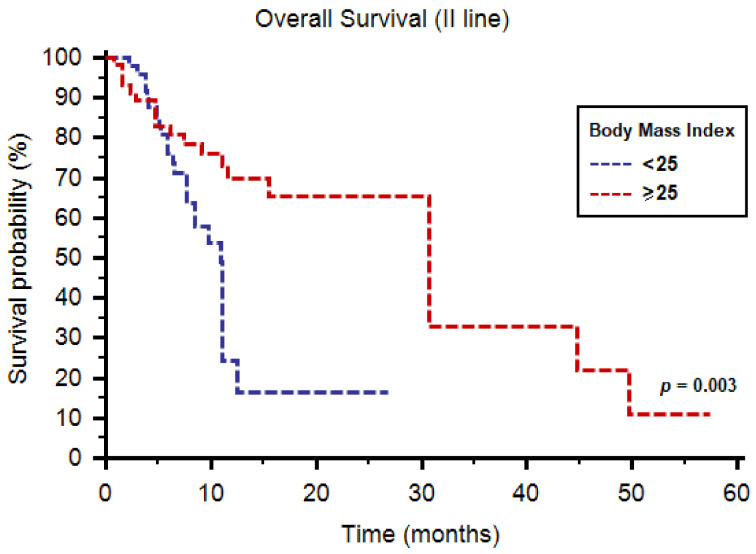
Overall survival of patients receiving cabozantinib as second-line therapy stratified by body mass index.

The median OS was 12.5 months (95%CI: 11.1–30.7) in all patients and was higher in the BMI ≥ 25 subgroup (30.7 months, 95%CI: 11.6–44.8, vs. 11.0 months, 95%CI: 9.7–11.2, *p* = 0.003, Figure 2).

In terms of 1-year OS rate, 76% of patients with BMI ≥ 25 were alive at 1 year, compared to 53% of patients with BMI < 25 (*p* = 0.019, Table 2). No significant differences were found concerning tumor response to cabozantinib as a second-line option (Table 3).

At multivariate analysis, IMDC prognostic group (hazard ratio (HR) = 2.09; 95%CI: 1.29–3.37, *p* = 0.003) and BMI (HR = 0.37; 95%CI: 0.20–0.68 *p* = 0.002) were significant predictors of PFS. Similarly, IMDC prognostic group (HR = 2.09; 95% CI, 1.29–3.37, *p* = 0.003) and BMI (HR = 0.38; 95%CI: 0.22–0.69, *p* = 0.046) were significantly correlated with OS at multivariate analysis.

**Figure 3:**
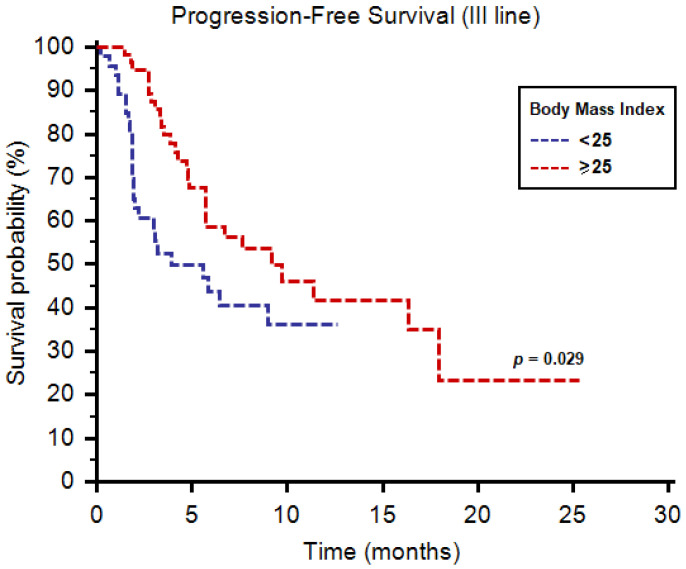
Progression-free survival of patients receiving cabozantinib as third-line therapy stratified by body mass index.

**Figure 4:**
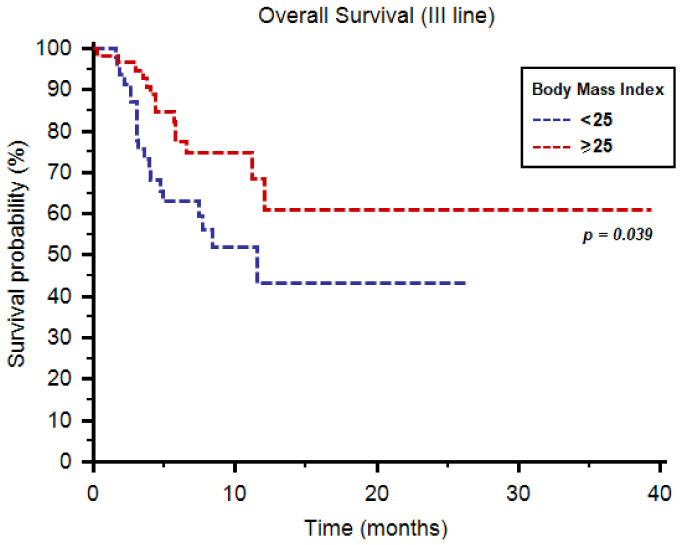
Overall survival of patients receiving cabozantinib as third-line therapy stratified by body mass index.

As third-line therapy, we registered median PFS and OS of 6.7 (95%CI: 4.9–18.0) and 39.4 months (95%CI: 11.2–39.4), respectively. Both median PFS (9.2 months, 95%CI: 4.8–16.3, vs. 3.9 months, 95%CI: 3.0–18.0, *p* = 0.029, Figure 3) and OS (39.4 months, 95%CI: 11.2–39.4, vs. 11.5 months, 95%CI: 4.9–11.5, *p* = 0.039, Figure 4) were longer in patients with BMI ≥ 25. One-year OS rate was 28% and 11% in patients with BMI ≥ 25 vs. <25, respectively (*p* = 0.045, Table 4). Similarly to second-line use of cabozantinib, no differences were reported in terms of tumor response to third-line cabozantinib (Table 4).

For both PFS (HR = 0.52; 95% CI, 0.32–0.95, *p* = 0.020) and OS (HR = 0.32; 95% CI, 0.16–0.695, *p* = 0.002) BMI was a significant predictor at multivariate analysis. Number of metastatic sites (≥2 vs. <2) was significantly associated with PFS (HR = 1.35; 95% CI, 1.04–1.74, *p* = 0.021) at multivariate analysis, while age (≥70 y vs. <70 y) was significantly correlated with OS at univariate (HR = 0.34; 95% CI, 0.12–0.96, *p* = 0.042) but not at multivariate analysis (HR = 0.70; 95% CI, 0.51–1.05, *p* = 0.074).

## 4. Discussion

The search for prognostic or predictive factors in patients with advanced RCC has become even more essential due to the evidence that immune combos could perform differently based on Memorial Sloan Kettering Cancer Center (MSKCC) risk stratification [15,16,17]. Interestingly, while treatments with nivolumab plus ipilimumab [15] or avelumab [16] do consider patient weight for a drug dose calculation, cabozantinib and other TKIs do not consider this parameter. In this view, we focused on the eventual role of BMI in RCC patients treated with cabozantinib. Our results clearly show that BMI strongly correlates with the outcome of patients treated with second- or third-line cabozantinib. Interestingly, no differences were reported in terms of histopathological and clinical features (i.e., number or type of metastatic sites and IMDC criteria) as well as tumor response. Our data are in line with those published by Martini et al. [18], who reported no differences in terms of adverse events between obese and non-obese patients receiving cabozantinib for advanced RCC.

Together with the published results on the correlation between obesity and response to immunotherapy [19], our results support a prognostic but not predictive role for BMI in RCC patients, even if the results in patients with BMI < 25 receiving cabozantinib as third-line therapy (median PFS = 3.7 months) seem to merit careful consideration. Nevertheless, our study presents several limitations (e.g., the lack of data on causes of death, comorbidities, and concomitant medications), mainly due to the biased characteristic of retrospective studies, and should be confirmed by prospective clinical studies.

As reported above, the “obesity paradox” in RCC patients receiving immunotherapy has been partially clarified by the evidence of different signatures and tumor-infiltrating immune cells between obese and non-obese subjects [8]. A potential explanation for the role of being overweight and obese in this setting of antiangiogenic therapies may be provided by the emerging data on the role of adipocytes in renal carcinogenesis. Indeed, adipose microenvironment can regulate the proliferation and migration of tumoral and non-tumoral human renal epithelial cells [20]. Increased levels of leptin could enhance the invasive potential of renal epithelial cell lines and could modulate the progression of the disease [21,22]. Furthermore, higher hypoxia-induced factor (HIF)-1 levels (which stimulates angiogenesis by deregulating the production of tumor necrosis factor-α, VEGF, and angiopoietin) have been reported in the adipose tissue of obese patients [23]. Moreover, adiponectin levels are higher in non-obese subjects [24], and exposure of RCC cell lines to adiponectin inhibits the secretion of VEGF [25].

## 5. Conclusions

We showed that a BMI ≥ 25 correlates with longer survival in patients receiving cabozantinib. BMI can be easily assessed and monitored during patients’ management, supporting its role as a tool for the decision-making process of advanced RCC. Further prospective studies should be provided in order to validate BMI among RCC prognostic criteria.

## Figures and Tables

**Table 1:** Patient demographic and disease characteristics. BMI: body mass index; IMDC: International Metastatic Renal Cell Carcinoma Database Consortium.

Patients	Overall 224 (%)	BMI ≥ 25 119 (%)	BMI < 25 105 (%)	*p*
**Gender**				0.657
Male	160 (71)	87 (73)	73 (70)	
Female	64 (29)	32 (27)	32 (30)	
**Age, years (y)**	63	63	63	-
Range	25–86	31–86	25–85	
**Karnofsky performance status** Score > 70	211 (94)	114 (96)	97 (92)	0.421
**Metastatic at diagnosis**	114 (51)	57 (48)	57 (54)	0.412
**Past nephrectomy**	173 (77)	92 (77)	81 (77)	0.897
**Clear cell histology**	193 (86)	99 (83)	94 (90)	0.240
**IMDC risk stratification**				0.219
Favorable risk	50 (22)	29 (24)	21 (20)	
Intermediate risk	134 (60)	65 (55)	69 (66)	
Poor risk	40 (18)	25 (21)	15 (14)	
**Common sites of metastasis**				
Lung	145 (65)	77 (65)	68 (65)	0.896
Lymph nodes	115 (51)	63 (53)	52 (50)	0.706
Bone	63 (28)	34 (29)	29 (28)	0.993
Liver	42 (19)	15 (13)	20 (19)	0.254
Brain	17 (8)	7 (6)	10 (10)	0.439
**≥2 Metastatic sites**	135 (60)	69 (58)	66 (63)	0.458

**Table 2:** Drug distribution and response to cabozantinib.

Patients	Overall	BMI ≥ 25	BMI < 25	*p*
	224 (%)	119 (%)	105 (%)	
**First-line therapy**				0.979
Sunitinib	121 (54)	64 (54)	57 (54)	
Pazopanib	73 (33)	38 (32)	35 (33)	
Immunocombinations	9 (4)	5 (4)	4 (4)	
Other	21 (9)	12 (10)	9 (9)	
**Second-line therapy**				0.862
Cabozantinib	113 (50)	62 (52)	51 (49)	
Nivolumab	89 (40)	46 (39)	43 (41)	
Other	22 (10)	11 (9)	11 (10)	
**Third-line therapy**				0.982
Cabozantinib	111 (50)	57 (48)	54 (51)	
Nivolumab	36 (16)	19 (16)	17 (16)	
Other	15 (7)	8 (7)	7 (6)	
**Response to 2nd-line cabozantinib**				0.916
CR/PR	31 (27)	18 (15)	13 (12)	
SD	54 (48)	29 (26)	25 (22)	
PD	28 (25)	15 (13)	13 (12)	
**Response to 3rd-line cabozantinib**				0.127
CR/PR	32 (29)	18 (16)	14 (13)	
SD	37 (33)	14 (13)	23 (20)	
PD	42 (38)	25 (17)	17 (21)	
**1y-OS (second-line cabozantinib)**	73 (65)	47 (76)	27 (53)	**0.019**
**1y-OS (third-line cabozantinib)**	22 (20)	16 (28)	6 (11)	**0.045**

CR: complete response; PR: partial response; SD: stable disease; PD: progressive disease. In bold statistically significant values.

**Table 3:** Univariate and multivariate analyses of predictors of progression-free survival and overall survival in patients treated with cabozantinib as second-line therapy.

**PFS**	**Univariate Cox Regression**		**Multivariable Cox Regression**	
	**HR (95%CI)**	***p*-Value**	**HR (95%CI)**	***p*-Value**
Age (≥70 y vs. <70 y)	1.31 (0.78–2.21)	0.306		
Gender (M/F)	1.17 (0.69–2.00)	0.563		
Number of metastatic sites	0.99 (0.78–1.27)	0.930		
Lung metastases	0.86 (0.59–1.47)	0.783		
Liver metastases	1.42 (0.77–2.51)	0.459		
Bone metastases	1.99 (1.08–3.18)	0.112		
IMDC prognostic group	2.08 (1.26–3.44)	0.004	2.09 (1.29–3.37)	**0.003**
BMI (≥25 vs. <25)	0.90 (0.84–0.97)	0.005	0.37 (0.20–0.68)	**0.002**
**OS**	**Univariate Cox Regression**		**Multivariable Cox Regression**	
	**HR (95%CI)**	***p*-Value**	**HR (95%CI)**	***p*-Value**
Age (≥70 y vs. <70 y)	0.78 (0.41–1.49)	0.459		
Gender (M/F)	1.46 (0.80–2.67)	0.219		
Number of metastatic sites	1.08 (0.81–1.44)	0.613		
Lung metastases	0.82 (0.51–1.33)	0.424		
Liver metastases	1.39 (0.80–2.39)	0.244		
Bone metastases	2.11 (1.32–3.40	0.051		
IMDC prognostic group	1.85 (1.02–3.34)	0.042	2.09 (1.29–3.37)	**0.003**
BMI (≥25 vs. <25)	0.90 (0.83–0.98)	0.018	0.38 (0.22–0.69)	**0.046**

BMI: body mass index; CI: confidence interval; HR: hazard ratio; OS: overall survival; PFS: progression-free survival; in bold statistically significant values.

**Table 4:** Univariate and multivariate analyses of predictors of progression-free survival and overall survival in patients treated with cabozantinib as third-line therapy.

**PFS**	**Univariate Cox Regression**		**Multivariable Cox Regression**	
	**HR (95%CI)**	***p*-Value**	**HR (95%CI)**	***p*-Value**
Age (≥70 y vs. <70 y)	0.59 (0.31–1.11)	0.102		
Gender (M/F)	0.86 (0.46–1.61)	0.645		
Number of metastatic sites	1.40 (1.10–1.80)	0.007	1.35 (1.04–1.74)	**0.021**
Lung metastases	0.86 (0.67–1.91)	0.672		
Liver metastases	1.75 (0.84–2.88)	0.594		
Bone metastases	1.87 (0.96–3.98)	0.317		
IMDC prognostic group	1.18 (0.76–1.82)	0.458		
BMI (≥25 vs. <25)	0.55 (0.32–0.95)	0.031	0.52 (0.32–0.95)	**0.020**
**OS**	**Univariate Cox Regression**		**Multivariable Cox Regression**	
	**HR (95%CI)**	***p*-Value**	**HR (95%CI)**	***p*-Value**
Age (≥70 y vs. <70 y)	0.34 (0.12–0.96)	0.042	0.70 (0.51–1.05)	0.074
Gender (M/F)	1.12 (0.52–2.39)	0.776		
Number of metastatic sites	1.29 (0.94–1.76)	0.120		
Lung metastases	0.91 (0.64–1.57)	0.458		
Liver metastases	1.82 (0.73–2.86)	0.632		
Bone metastases	1.91 (1.08–3.41)	0.351		
IMDC prognostic group	1.51 (0.87–2.64)	0.144		
BMI (≥25 vs, <25)	0.35 (0.18–0.70)	0.003	0.32 (0.16–0.65)	**0.002**

BMI: body mass index; CI: confidence interval; HR: hazard ratio; OS: overall survival; PFS: progression-free survival; in bold statistically significant values.

## Data Availability

The data presented in this study are available on request from the corresponding author. The data are not publicly available due to privacy restrictions.
